# BADAN-conjugated β-lactamases as biosensors for β-lactam antibiotic detection

**DOI:** 10.1371/journal.pone.0241594

**Published:** 2020-10-30

**Authors:** Ho-Wah Au, Man-Wah Tsang, Yu Wai Chen, Pui-Kin So, Kwok-Yin Wong, Yun-Chung Leung

**Affiliations:** 1 Department of Applied Biology and Chemical Technology, State Key Laboratory of Chemical Biology and Drug Discovery, The Hong Kong Polytechnic University, Hung Hom, Kowloon, Hong Kong, China; 2 Lo Ka Chung Research Centre for Natural Anti-Cancer Drug Development, The Hong Kong Polytechnic University, Hung Hom, Kowloon, Hong Kong, China; Consiglio Nazionale delle Ricerche, ITALY

## Abstract

β-Lactam antibiotic detection has significant implications in food safety control, environmental monitoring and pharmacokinetics study. Here, we report the development of two BADAN-conjugated β-lactamases, E166Cb and E166Cb/N170Q, as sensitive biosensors for β-lactam antibiotic detection. These biosensors were constructed by coupling an environment-sensitive BADAN probe onto location 166 at the active site of the PenP β-lactamase E166C and E166C/N170Q mutants. They gave fluorescence turn-on signals in response to β-lactam antibiotics. Molecular dynamics simulation of E166Cb suggested that the turn-on signal might be attributed to a polarity change of the microenvironment of BADAN and the removal of the fluorescence quenching effect on BADAN exerted by a nearby Tyr-105 upon the antibiotic binding. In the detection of four β-lactams (penicillin G, penicillin V, cefotaxime and moxalactam), both E166Cb and E166Cb/N170Q delivered signal outputs in an antibiotic-concentration dependent manner with a dynamic range spanning from 10 nM to 1 μM. Compared to E166Cb, E166Cb/N170Q generally exhibited more stable signals owing to its higher deficiency in hydrolyzing the antibiotic analyte. The overall biosensor performance of E166Cb and E166Cb/N170Q was comparable to that of their respective fluorescein-modified counterparts, E166Cf and E166Cf/N170Q. But comparatively, the BADAN-conjugated enzymes showed a higher sensitivity, displayed a faster response in detecting moxalactam and a more stable fluorescence signals towards penicillin G. This study illustrates the potential of BADAN-conjugated β-lactamases as biosensing devices for β-lactam antibiotics.

## Introduction

β-Lactam antibiotics have been important therapeutics in human and animal medications for decades [1]. Owing to their high cost-effectiveness in treating bacterial infections, they have been nowadays the most frequently used antibiotics worldwide [2, 3]. With the extensive application of these drugs, concerns have been raised on the β-lactam antibiotic contamination in food and environment which leads to a health risk on hypersensitive individuals, a spread of antibiotic resistance and technical difficulties in fermentation [4–9]. In addition, the effectiveness of the once-dependable β-lactam antibiotics has been greatly challenged by a rapid emergence of the bacterial resistance associated with the abusive and indiscriminate antibiotic uses [6–8]. Thus, there has been a rising number of untreatable infectious diseases in recent years, posing a global health crisis [6–8]. To tackle these problems, β-lactam antibiotic level in food and biological samples (e.g. plasma) must be stringently controlled and its monitoring has been an essential task in food industry and clinical practice.

Presently, microbial inhibition assays and rapid tests are two main conventional methods for β-lactam antibiotic detection [4]. On one hand, microbial inhibition assays (e.g. Delvotest SP) are methods based on the inhibition of bacterial growth by the antibiotic residue in the sample [10–12]. They are simple and inexpensive but time-consuming for microbial growth, and lack of selectively and sensitivity. They are often used for the nondiscriminatory detection of the antibiotic. On the other hand, rapid tests (e.g. Parallux and Charm Test) are semi-quantitative analyses relied on the interaction between antibody or receptor protein and the antibiotic [13–15]. Because these tests are rapid, sensitive and specific, they are employed in fast screening of the β-lactam antibiotics. Apart from these two conventional approaches, analytical methods such as high-performance liquid chromatography (HPLC) and mass spectrometry (MS) have also been devised for β-lactam antibiotic detection [16, 17]. These qualitative methods provide a confirmatory examination of the drug’s authenticity and a precise determination of the antibiotic level. However, they require sophisticated instruments and tedious sample preparation, limiting their use as a final step for discriminatory examination of the antibiotic level.

To address the growing demands for efficient monitoring system for β-lactam antibiotics, our laboratory has previously developed a series of fluorescent β-lactamase-based biosensors [18–22]. With a fluorescein covalently attached at the active site’s entrance, the β-lactamases were turned into biosensors that give fluorescence turn-on signals towards the β-lactam antibiotics. Our previous results have showed that the fluorescein-attached β-lactamases enable rapid, sensitive and direct biosensing for β-lactam antibiotics and β-lactamase inhibitors [18–22]. Their utility in biosensing merits various aspects. First, it facilitates the food safety control by preventing the antibiotic contamination in food. This can reduce the health hazard of antibiotic allergy and antibiotic resistance by minimizing the exposure of the antibiotics to the hypersensitive individuals and the environment [23]. Second, it offers a pharmacokinetics monitoring of the medically prescribed β-lactam antibiotics [24, 25]. This surveillance provides valuable information for optimizing the antibiotic dosage, which prevents the overuse of β-lactam antibiotics. Third, it allows a specific screening of β-lactamase inhibitors, which are important therapeutics for restoring the effectiveness of β-lactam antibiotics in combating the β-lactamase-producing pathogenic bacteria [18, 19, 21]. Furthermore, it offers a real time fluorescence monitoring of the interaction between β-lactamases and their substrates/inhibitors. This provides a better understanding of the hydrolytic/inactivation mechanisms of the β-lactamases.

In this study, we aimed at improving our previous PenP-E166Cf and E166Cf/N170Q for a better antibiotic detection. These biosensors were prepared by attaching a fluorescein molecule onto site 166 at a flexible Ω-loop of the thermostable PenP β-lactamase E166C mutants. E166Cf/N170Q was modified from E166Cf by introducing a N170Q mutation. Because the N170Q mutation further reduced the degradation rate of the β-lactam analytes by the enzyme-based biosensor, E166Cf/N170Q showed an enhanced signal stability than its parental E166Cf. In this current work, we attempted to substitute the fluorescein of these biosensors with another fluorescent probe, BADAN (6-bromo-acetyl-2-dimethylamino-naphtalene), as the signal transducing component of the biosensor. Here, the E166C-based mutants were conjugated with a BADAN to give the E166Cb and E166Cb/N170Q respectively (S1 Fig). BADAN is a fluorescent solvatochromic dye [26]. Our rationale in exploiting BADAN is based on its high sensitivity to the microenvironment change. In addition, its small size (molecular size: 292.1) also makes it a favorable candidate as a reporter in our biosensor’s active site because this minimizes the perturbation to the enzyme’s structure and the steric hindrance to the analyte-binding event. In our previous study, PenP-E166Cb was created to investigate the relationship between the flexibility of the Ω-loop and substrate specificity of β-lactamase [27]. Our results showed that BADAN can experience the local environment changes upon binding with various cephalosporins and transduce them into fluorescence readout for probing the structural flexibility of the Ω-loop. In this current study, we moved on to explore the feasibility of E166Cb as a biosensing system for β-lactam antibiotics. Moreover, we also constructed an E166Cb/N170Q derivative as we expected that this preparation might give more stable signals than E166Cb.

## Material and methods

### Materials

BADAN was purchased from Molecular Probes Inc. (Eugene, OR, USA). Cefotaxime, moxalactam, penicillin G, penicillin V, kanamycin and lysozyme were purchased from Sigma (St. Louis, MO, USA). Tryptone and yeast extract used for preparing 2×TY medium were obtained from Oxoid (Nepean, Ontario, Canada). The chemical structures of BADAN and β-lactam antibiotics involved in this study were illustrated in S1 Fig.

### Production of PenP β-lactamases

#### Protein expression

PenP β-lactamase E166C and E166C/N170Q mutants were produced as previously described [22]. Briefly, a single colony of *E*. *coli* BL21(DE3) harboring the expression construct of maltose binding protein (MBP)-tagged PenP mutant was inoculated into 5 mL of 2×TY medium supplemented with 50 mg/mL kanamycin. The inoculum was cultivated at 37°C with a shaking at 300 rpm for 14 h. The overnight culture was then transferred into fresh 2×TY medium in a 1:100 ratio. The culture was incubated at 37°C with a 300 rpm-agitation. When the OD_600_ of the culture reached 0.7, protein expression was induced by adding 300 μM IPTG to the culture medium. Cells were further grown for 4 h at 37°C with shaking at 300 rpm, and then harvested by centrifugation at 6500 rpm, 4°C for 25 min. Cell pellet was kept at -80°C till purification.

#### Protein purification

Prior to purification, the cell pellet was resuspended in binding buffer (20 mM Tris-HCl containing 0.2 M sodium chloride and 1mM EDTA, pH 7.4). The cell suspension was then treated with 75 mg/mL lysozyme and incubated at 30°C for 45 min. After that, cell was lysed by sonication with 5 cycles of short pulses for 30 s on ice. The lysate was then centrifuged at 10,000 rpm for 1 h at 4°C. After that, supernatant was collected and clarified with a 0.45 μm syringe filter. A column packed with amylose affinity gel (New England Biolabs, Beverly, US) was equilibrated with the binding buffer and then loaded with the clarified cell lysate. It was washed with a 2.5 column volume of the binding buffer to remove the unbound molecules. Protein was eluted with a linear maltose gradient from 0 to 4 mM. Fractions containing the target protein was collected and concentrated by ultrafiltration. The sample was then dialyzed against 20 mM ammonium bicarbonate, lyophilized and stored at -20°C.

### Fluorescence labeling

To ensure a complete exposure of cysteine for the labeling reaction, the lyophilized β-lactamase cysteine mutant was reconstituted in 6 M guanidine hydrochloride to a concentration of 0.5 mg/mL and unfolded with stirring for 30 min at room temperature. Meanwhile, a stock solution of 20 mM BADAN was prepared by dissolving the chemical in dimethylformamide (DMF) at dark. Prior to labeling, the pH of the protein solution was adjusted to 7.5 with 0.2 M sodium hydroxide. To label the protein, a 10-fold molar excess of BADAN was added to the solution of the unfolded protein. Then the mixture was subjected to a 2 h-incubation at room temperature with stirring at the dark. Afterward, the mixture was buffer exchanged with 50 mM potassium phosphate immediately at 4°C to allow protein refolding and removal of excess fluorescent dye. BADAN-conjugated protein was then stored at -20°C.

### Electrospray ionization mass spectrometry (ESI-MS)

ESI-MS was performed on a quadrupole-time of flight mass spectrometer (Q-TOF2, Waters/Micromass, Milford, MA) coupled with an electrospray ionization (ESI) source. Protein samples in H2O/CH3CN (1:1 v/v) containing 0.5% formic acid (v/v) were infused into the ESI source at a flow rate of 5 μL/min. The acquisition *m/z* range was 600–1600. To determine the average molecular mass of E166Cb and E166Cb/N170Q, raw multiply charged spectra were de-convoluted by the MassLynx 4.0 Transform Program (Waters/Micromass, Milford, MA).

### Circular dichroism (CD)

Far-UV CD spectrometry was conducted on a Jasco 810 spectropolarimeter (Jasco International Co. Ltd., Tokyo, Japan). Protein samples were prepared in 5 mM potassium phosphate (pH 7.0) at a concentration of 150–200 μg/mL and then placed on the cuvette with a path length of 1.0 mm for CD analysis. CD spectra were collected by scanning the samples over the wavelength from 185 nm to 250 nm with a scan speed of 50 nm/min.

### Fluorescence measurements

All fluorescence measurements were conducted at 20°C in 50 mM potassium phosphate (pH 7.0) on a Perkin Elmer LS50B spectrofluorimeter (Perkin Elmer, Germany). Excitation wavelength was set at 380 nm for the BADAN-labeled samples. Time-dependent fluorescence traces were acquired at the emission wavelength of 500 nm. Both excitation and emission slit widths were 10 nm.

### Molecular dynamics (MD) simulations

Both apo- and substrate-bound protein models were based on the crystal structure of the *Bacillus licheniformis* PenP β-lactamase E166H mutant in complex with cephaloridine (PDB ID: 5GHY; A chain, with ligand removed). The apo-protein model of E166Cb was built by substituting His-166 with a BADAN-attached Cys-166 modified residue, in Coot and with the help of JLigand [28, 29]. The substrate-bound model was constructed similarly but with a penicillin G bound to Ser-70 of the β-lactamase, replacing the bound cephaloridine of 5GHY. The two modified residues were parameterized using the Automated Topology Builder (https://atb.uq.edu.au/) [30]. MD simulations of E166Cb with and without penicillin G were performed with the same procedures using GROMACS version 5.0 (double precision) [31] using the GROMOS 54A7 force field. Initially, all protein atoms except those in the Ω-loop were restrained at 1000 kJ mol^-1^ nm^-2^. Steepest descent energy minimization in vacuum was performed to relieve bad geometries and contacts. The model was immersed in a cubic solvent box with 1.0 nm thick walls. Single Point Charge (SPC) water model was used, followed by an addition of 150 mM ions to neutralize the overall charge of the protein. After that, the system was energy minimized again and then equilibrated in NVT ensemble (constant number, volume and temperature) for 100 ps and subsequently, in NPT ensemble (constant number, pressure and temperature), during which the positional restraints were gradually reduced from 1000 to 0.1 kJ mol^-1^ nm^-2^ in 8 steps of 100 ps each. The production run for 10 ns was performed without restraints. The structure and energies were saved every 10 ps. Simulation trajectories were visualized using the VMD program [32]. Structure of the models after performing MD was analyzed by inbuilt utilities of GROMACS, including rmsdist, gyrate, sasa and pairdist.

## Results and discussion

### Construction of E166Cb and E166Cb/N170Q

E166Cb and E166Cb/N170Q were built on the same protein framework of a PenP β-lactamase. In the biosensor construction, Glu-166 of the wild-type enzyme was substituted with a cysteine which provided a linker for the bio-conjugation with the thiol-reactive BADAN. Because it was the only cysteine in the E166C mutant, non-specific conjugation with BADAN to other sites could be avoided. In addition, E166C mutation remarkably impaired the catalytic activity of the β-lactamase with a restoration of the substrate binding properties [22]. Hence, a hydrolytically competent β-lactamase was converted into a “substrate-binding protein”. To make the biosensor scaffold more mimic a pure binding protein, a N170Q mutation was introduced onto the E166C mutant to further suppress the catalytic activity [22]. Through a single step of coupling reaction, the biosensors were generated through the thiol chemistry between the bromoacetyl moiety of BADAN and the -SH group of the enzyme’s cysteine at position 166 (S1 Fig). The authenticity of E166Cb and E166Cb/N170Q was checked by ESI-MS. The measured masses of E166Cb and E166Cb/N170Q were 72778.48 ± 1.01 Da and 72792.4 ± 3.78 Da respectively (S2 Fig). These values were consistent with their corresponding calculated masses which were 72778 Da for E166Cb and 72792 Da for E166Cb/N170Q. This indicates that both mutants were conjugated with BADAN in a 1:1 stoichiometry. According to CD analysis (S3 Fig), both E166Cb and E166Cb/N170Q gave a similar spectrum with a positive peak at 195 nm and a negative shoulder between 205–225 nm. The intensity and shape of their CD signals were similar as those displayed by their unlabeled parental enzymes. There was no noticeable spectral change between the unlabeled and BADAN-labeled enzymes. This data reveals a proper refolding of the proteins and also the preservation of overall enzymes’ secondary structures after the labeling reaction.

### Fluorescence properties of E166Cb and E166Cb/N170Q

**Fig 1:**
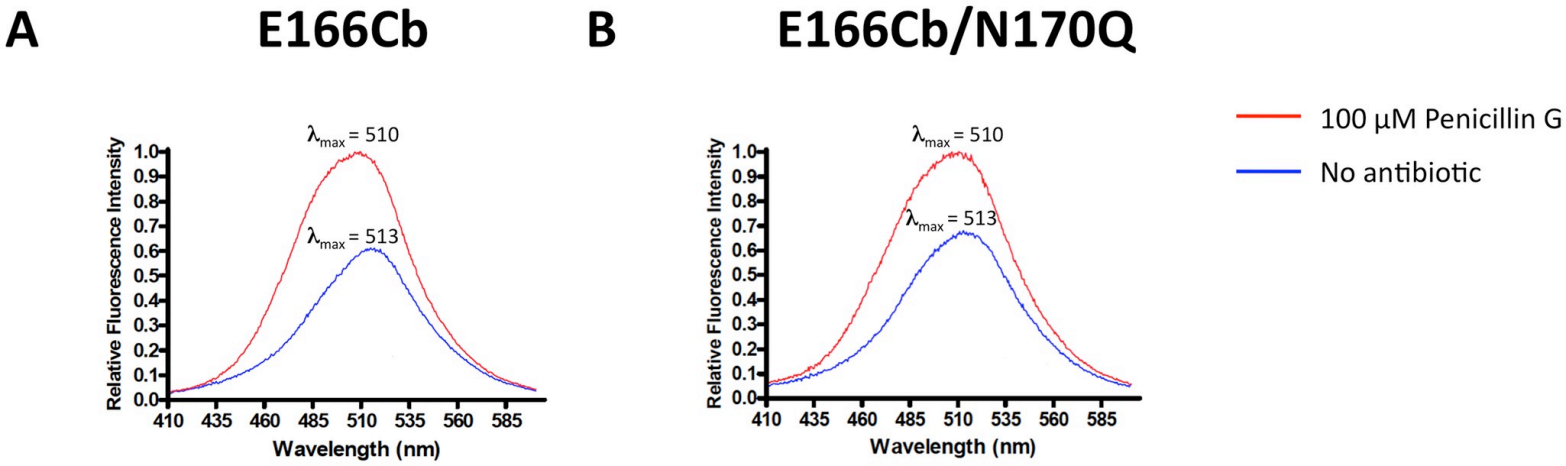
Fluorescence scanning of BADAN-conjugated β-lactamases, (A) E166Cb and (B) E166Cb/N170Q. Spectra were obtained by scanning 0.1 μM labeled enzyme in 50 mM potassium phosphate buffer (pH 7.0) with an excitation at 380 nm. Blue line: enzyme without penicillin G; Red line: enzyme with the addition of 100 μM penicillin G.

Fig 1 illustrates the fluorescence spectral properties of the BADAN-conjugated β-lactamases with and without penicillin G. Upon an excitation at 380 nm, both E166Cb and E166Cb/N170Q showed their emission maximum at 513 nm in their apo-forms. In the presence of penicillin G, they demonstrated a fluorescence enhancement (E166Cb: 39% increase; E166Cb/N170Q: 32.4% increase) with a blue shift of their emission maximum from 513 nm to 510 nm. This wavelength shift suggested that the environment of BADAN turned slightly non-polar in the presence of the antibiotic.

### Molecular dynamics of E166Cb with and without β-lactam antibiotic

To elucidate the fluorescence mechanism of the biosensors, MD simulations of E166Cb with and without penicillin G were conducted to predict the substrate-induced conformational and local environmental changes of BADAN. For each 10-ns MD trajectory, the root-mean-square deviation (RMSD) and the radius of gyration (Rg) of the protein were assessed to evaluate the compactness and stability of the protein models. For both apo- and substrate-bound E166Cb, a relatively stable value of RMSD was obtained after 1 ns whereas a steady value of Rg was maintained until 9 ns (S4 Fig). Thus, the data within the MD times from 1 to 9 ns was used for our analysis.

**Fig 2:**
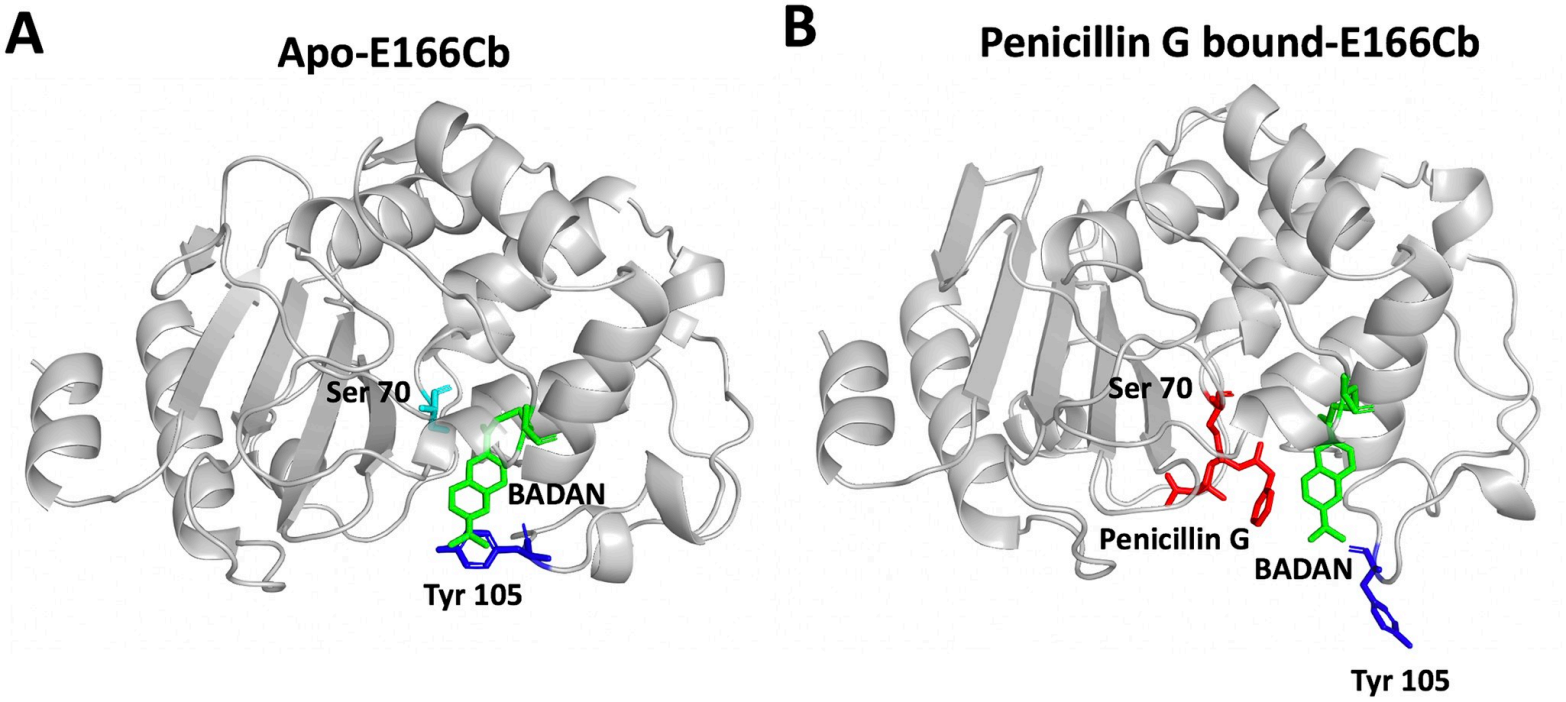
Models of E166Cb with and without penicillin G. (A) In the apo-E166Cb model, BADAN (green) was slightly buried in the active site pocket and stayed close to Tyr-105 (blue). Its naphthalene core was stacking on the aromatic ring of the tyrosine. (B) In the presence of penicillin G (red), BADAN moved slightly towards the aqueous environment and it was spatially separated from Tyr-105.

As shown in the apo-E166Cb model (Fig 2A), the covalently tethered BADAN on the flexible Ω-loop was slightly hidden in the active site cavity. Noticeably, it was in close proximity to Tyr-105 which was situated on another loop region at the active site’s entrance. Along the MD trajectory, the naphthalene moiety of BADAN often stacked with the aromatic ring of Tyr-105. In the penicillin G-bound state (Fig 2B), there were changes in conformation and the surrounding environment of the BADAN. First, the fluorophore was in close proximity to the benzene ring of the penicillin G in the active site pocket. Moreover, it reoriented slightly away from the hydrophobic active site towards the solvent. The total solvent accessible areas of BADAN in the absence and presence of penicillin G were 77.8 ± 23.4 Å and 115.9 ± 23.8 Å respectively (S5A Fig). Furthermore, BADAN lost the close contact to Tyr-105. The average distance between the mass centers of BADAN and Tyr-105, in the substrate-bound simulation, was 10.3 ± 3.0 Å (Fig 2B). This is two times of that of the apo-E166Cb model (5.2 ± 1.1 Å) (S5B Fig). These results indicate that the solvent exposure of BADAN increased slightly in the presence of penicillin G. On the other hand, the proximity of the benzyl group of penicillin G increased the hydrophobicity of the environment of BADAN. We postulated that overall, the local environment became slightly less polar, which correlated to the blue shifting of the emission maximum in penicillin-G bound E166Cb (Fig 1). In addition, a relief of the BADAN from the quenching effect by a neighboring amino acid (Tyr-105) possibly accounted for the observed enhancement of E166Cb’s fluorescence intensity. A similar fluorescence mechanism was also reported by another study which demonstrated the fluorescence quenching of a covalently linked BADAN in cytochrome P450 by a nearby tryptophan [33].

We expect that the cephalosporin-induced conformational change might be similar to the change observed in penicillin G-bound model. According to the substrate-bound models of E166Cb (S6 Fig), the thiazolidine ring in penicillin G and dihydrothiazine core of cephaloridine (a cephalosporin) showed a similar orientation in the active site pocket. In addition, neither of these rings is in close contact with BADAN. Therefore, it is unlikely that the thiazolidine ring in penicillin or dihydrothiazine ring in cephalosporin affects the fluorescence of BADAN. Indeed, it is the R1 side chain of the antibiotics positioning close to BADAN. Thus, the R1 side chain possibly plays a role in affecting the environment polarity of the fluorescent probe, which influences the fluorescence of the biosensor.

We speculated that the prediction made from E166Cb can also apply to E166Cb/N170Q because N170Q mutation is a conservative change and does not change the overall polarity of the active site [34]. In addition, our fluorescence data also illustrated that E166Cb/N170Q possessed similar fluorescence spectral properties as E166Cb (Fig 1).

### Antibiotic detection by E166Cb and E166Cb/N170Q

**Fig 3:**
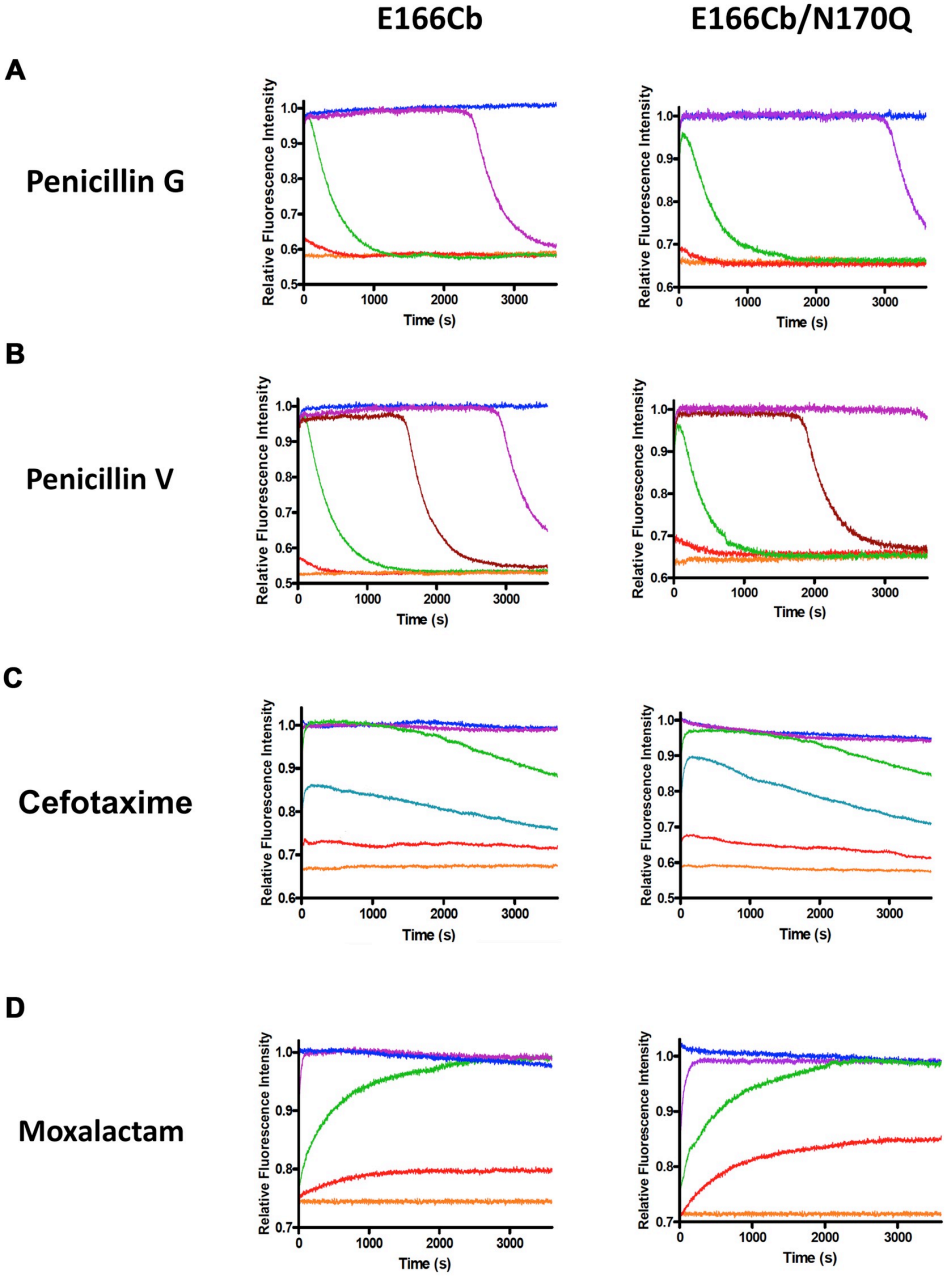
Fluorescence detection of β-lactam antibiotics by BADAN-conjugated β-lactamases, E166Cb and E166Cb/N170Q. Fluorescence traces were acquired after adding 0.1 μM labeled enzyme with (A) penicillin G, (B) penicillin V, (C) cefotaxime and (D) moxalactam in 50 mM potassium phosphate buffer (pH 7.0). Antibiotic concentrations: 0 (orange), 10 nM (red), 50 nM (cyan), 0.1 μM (green), 0.5 μM (brown), 1μM (purple) and 10 μM (blue). Fluorescence measurements were conducted several times with similar results. Representative traces were illustrated.

To demonstrate the capability of the BADAN-conjugated β-lactamases in detecting antibiotics, fluorescence behaviors of E166Cb and E166Cb/N170Q towards various β-lactams, including two penicillins (penicillin G and penicillin V) and two cephalosporins (cefotaxime and moxalactam), were examined. Fluorescence profiles were determined by monitoring the change upon the addition of the antibiotic at 500 nm as a function of time (Fig 3). In general, after mixing with the antibiotic, fluorescence of these enzymes first rose and then reached a peak level. The magnitude of the enhancement was dependent on the antibiotic concentration. The signal leveled off after attaining its maximal level and eventually vanished due to the substrate hydrolysis by the β-lactamase biosensors.

To evaluate the biosensors for antibiotic detection, parameters including response time, maximum signal change, signal duration and detection limit were determined from the time-dependent fluorescence traces (Fig 3) and calibration curves were constructed by plotting the percentage change in the peak fluorescence as a function of antibiotic concentration (S7 Fig). The biosensing performance of the BADAN-conjugated β-lactamases was summarized and compared with that of their fluorescein-labeled counterparts (Table 1).

**Table 1:** Biosensor performance of fluorescent β-lactamases.

		E166Cb	E166Cb/N170Q	E166Cf^a^	E166Cf/N170Q^a^
Response time (s)^b^	Penicillin G	62.5	66.7	ND^g^	ND
	Penicillin V	66.7	66.7	ND	ND
	Cefotaxime	62.5	133.3	ND	ND
	Moxalactam	62.5	200	688	600
Maximum signal change (%)^c^	Penicillin G	38.8	34.4	35.6	34
	Penicillin V	46.3	34.4	30	33
	Cefotaxime	32	41.1	22.5	20
	Moxalactam	24.6	27.5	25.8	29.1
Signal duration (s)^d^	Penicillin G	2000	2933	1867	2353
	Penicillin V	2866.7	3400	2474	> 3600; NM
	Cefotaxime	> 3600; NM^f^	> 3600; NM	> 3600; NM	> 3600; NM
	Moxalactam	> 3600; NM	> 3600; NM	> 3600; NM	> 3600; NM
Detection limit (nM)^e^	Penicillin G	10	10	10	10
	Penicillin V	10	10	10	10
	Cefotaxime	10	10	10	10
	Moxalactam	10	10	10	10

^a^ Values are cited from our previous data. [22]

^b^ Response time (s): time taken by the biosensor to give the peak fluorescence signal after adding 1 μM antibiotic.

^c^ Maximum signal change (%): the peak fluorescence level of the biosensor in response to 1 μM antibiotic.

^d^ Signal duration (s): the longevity of the fluorescence signal delineated by the time at which the maximum signal starts to decline. It is determined in the presence of 1 μM antibiotic.

^e^ Detection limit (nM): the lowest antibiotic level that can be sensed by the biosensor.

^f^ NM: not measured.

^g^ ND: not detectable because the response time is too short to be recorded by the fluorometer.

The linear dynamic range of detection of all fluorescent β-lactamases was from 10 nM to 1 μM (S7 Fig). For the four tested antibiotics, the saturating concentration for all biosensors to give a maximum signal was 1 μM. Increasing the substrate concentration to 10 μM did not modulate any further increase in the fluorescence intensity. Like the fluorescein-labeled enzymes, E166Cb and E166Cb/N170Q can detect the tested antibiotics at the concentration as low as 10 nM.

Maximum change of the fluorescence signal was recorded when the labeled β-lactamase was saturated with 1 μM antibiotic. In brief, BADAN-labeled biosensors demonstrated a wider range of the maximum signal change than the fluorescein-modified enzymes towards all antibiotics (E166Cb: 24.6–46.3%; E166Cb/N170Q: 27.5–41.1%; E166Cf: 22.5–35.6%; E166Cf/N170Q: 20–34%) (Table 1 and S7 Fig). Among all biosensors, E166Cb showed the largest change in the peak fluorescence in the detection of penicillins whereas E166Cb/N170Q illustrated the greatest maximum change towards cephalosporins. In addition, the BADAN-based biosensors generally showed a steeper slope than the fluorescein-labeled proteins in the calibration curves of antibiotic detection (S7 Fig). This implies that the BADAN-labeled β-lactamases showed higher affinities towards the tested antibiotics than their fluorescein-modified counterparts. Taken together, the BADAN-conjugated β-lactamases had a higher sensitivity than the fluorescein sensors in the antibiotic detection.

In this study, we define response time as the time taken by the labeled β-lactamase to react with a saturating amount (1 μM) of antibiotic to give a maximum signal. According to our results, E166Cb displayed a peak signal towards the four tested antibiotics within 60–70 s. Compared with E166Cb, E166Cb/N170Q took a similar time to have its fluorescence reaching the peak level upon the addition of penicillins but a doubled time with cephalosporins. Except the case of moxalactam, BADAN-conjugated enzymes showed a slower response time towards the antibiotics than their fluorescein-modified counterparts.

Signal stability is reflected by the duration of the signal at the saturating concentration (1 μM). For the detection of cephalosporins, all fluorescent enzymes displayed a stable signal within the 1 h monitoring timeframe. On the contrary, for sensing penicillins, both BADAN- and fluorescein-labeled E166C/N170Q can maintain a more sustainable signal than the labeled E166C mutant. As demonstrated in our previous study, the enhanced signal stability was resulted from the further catalytic impairment by a N170Q mutation [22]. With an exception of detecting penicillin V by E166Cb/N170Q, the signal duration of the BADAN-conjugated enzymes was generally longer than that of the fluorescein-modified counterparts in detecting penicillin-type antibiotics.

### Implications of BADAN-labeled β-lactamases

In this study, we illustrated that through an active site modification with a BADAN, catalytically deficient mutants of a PenP β-lactamase could be engineered into biosensors for fluorescent detection of the β-lactam antibiotics. In our E166C-based platforms, a BADAN molecule was strategically placed at the active site entrance and close to a tyrosine quencher. Antibiotic-binding event was converted into a fluorescence turn-on signal as it changed the environment polarity of BADAN and also relieved BADAN from the fluorescence quenching by the nearby tyrosine. Our study showed that rather than having a large analyte-induced conformational change of the fluorophore, the quenching system between BADAN and a neighboring tyrosine was effective enough for signal generation. Hence, we foresee that our active site labeling approach may be applicable to other enzymes for biosensor development. The next step of this study will be to develop a high-throughput antibiotic detection platform with the BADAN-conjugated β-lactamases for biological samples like milk and plasma. In addition, apart from the detection of β-lactam antibiotics, studies are underway to employ these sensors for screening the β-lactamase inhibitors. As β-lactamase inhibitors are often co-administrated with β-lactam antibiotics in the bacterial infection treatments [35, 36], we envision that the BADAN-conjugated β-lactamases may also be used for determining the effective regime of β-lactam antibiotic/β-lactamase inhibitor combinations.

## Conclusion

In conclusion, the BADAN-labeled β-lactamases, E166Cb and E166Cb/N170Q, showed the potential as efficient systems for β-lactam detection. Not only demonstrating a rapid detection, they also illustrated an improved sensitivity than their fluorescein-modified counterparts. Further work will be focus on the application of the BADAN-labeled β-lactamases as high throughput screening tools for food and clinical specimens.

## Supporting information

S1 Fig Chemicals used in this study. (A) BADAN and its bioconjugation to E166C β-lactamase via the formation of a thiol ester bond to give E166Cb; (B) β-lactam antibiotics.
(DOCX)

S2 Fig Mass spectra of (A) E166Cb and (B) E166Cb/N170Q. Peak A refers to the major species of the sample. E166Cb: calculated mass = 72778 Da and measured mass = 72778.48 ± 1.01 Da; E166Cb/N170Q: calculated mass = 72792 Da and measured mass = 72792.4 ± 3.78 Da. Calculated masses of E166Cb and E166Cb/N170Q were deduced from the sequence of the corresponding protein with the addition of one BADAN molecule.
(DOCX)

S3 Fig Far-UV CD spectra of (A) E166C and (B) E166C/N170Q before and after labeling with BADAN. Red line: before labeling; Green line: after labeling.
(DOCX)

S4 Fig Properties of the protein structure in the MD stimulation. (A) Root-mean-square deviation (RMSD) calculated using the energy-minimized structure after NVT and NPT equilibrations (npt8.gro) as the reference; (B) Radius of gyration (Rg); Red line: apo-E166Cb; Blue line: penicillin G-bound E166Cb.
(DOCX)

S5 Fig Properties of BADAN in the MD trajectories. (A) Accessible surface area of BADAN; (B) Distance of the mass center between BADAN and Tyr-105; Red line: apo-E166Cb; Blue line: penicillin G-bound E166Cb. Error bar of each data point represents the mean ± standard derivation.
(DOCX)

S6 Fig The substrate-bound models of E166Cb with either penicillin G or cephaloridine. (A) Structures of penicillin and cephalosporin; (B) An overlay of penicillin G and cephaloridine in the active site of E166Cb. Active site Ser-70: cyan; BADAN: green; penicillin G: red; Cephaloridine: yellow.
(DOCX)

S7 Fig Calibration curves of β-lactam antibiotic detection. (A) Penicillin G; (B) Penicillin V; (C) Cefotaxime; (D) Moxalactam; E166Cb: red line; E166Cb/N170Q: blue line; E166Cf: green line; E166Cf/N170Q: cyan line. Curves of E166Cf and E166Cf/N170Q were adapted from our previous data [22] (https://pub.acs.org/doi/10.1021/acsomega.9b02211) and reprinted in part with permission from American Chemical Society (ACS). A further permission of this data should be directed to the ACS.
(DOCX)
