# Supplementary material for: BADAN-conjugated β-lactamases as biosensors for β-lactam antibiotic detection
Source: PLoS One. 2020 Oct 30;15(10):e0241594. doi: 10.1371/journal.pone.0241594 (PMC7598492; doi:10.1371/journal.pone.0241594)
Supplement: S1 Fig — (A) BADAN and its bioconjugation to E166C β-lactamase via the formation of a thiol ester bond to give E166Cb; (B) β-lactam antibiotics. (DOCX) [file pone.0241594.s001.docx]

**
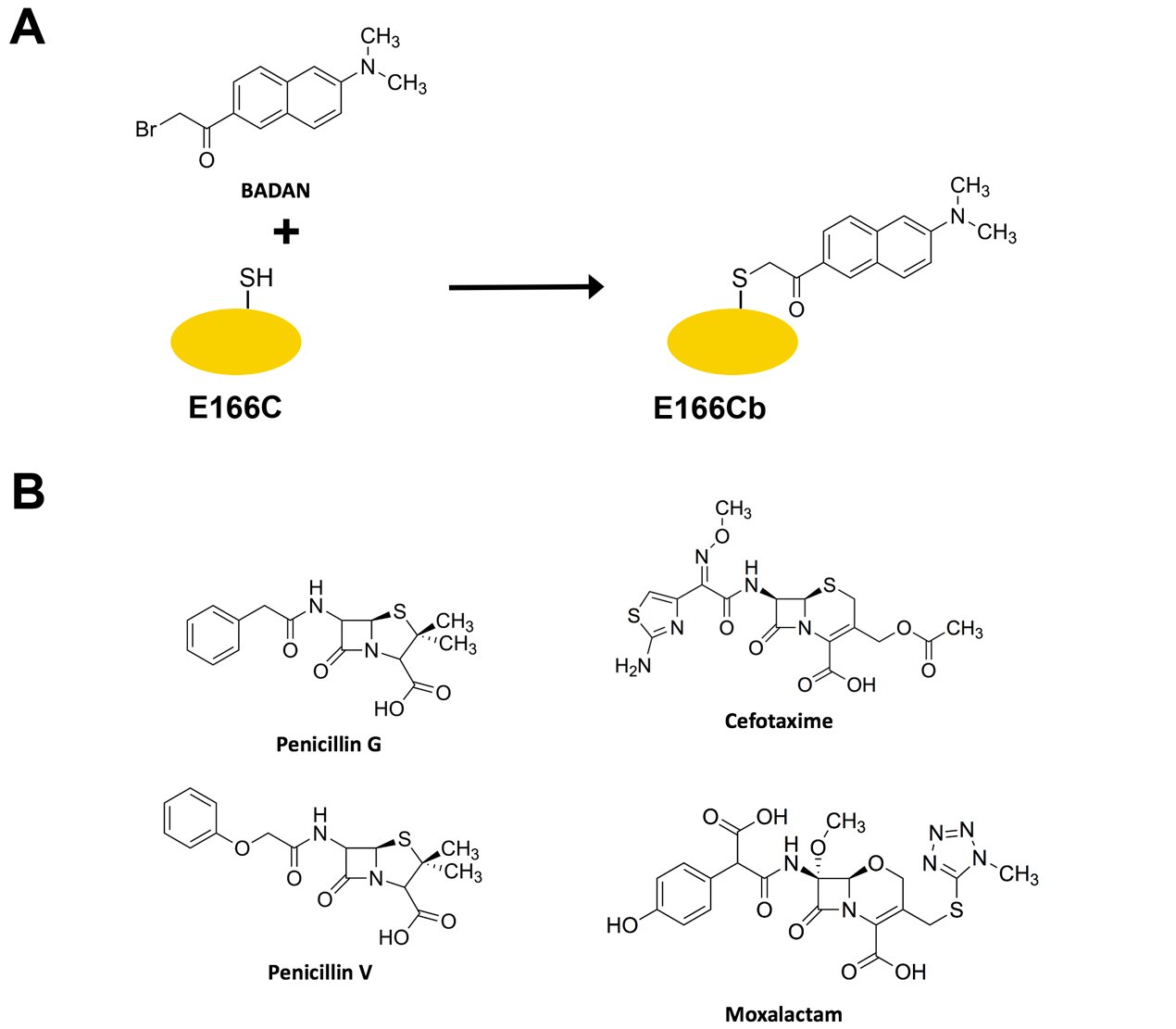
**

**S1 Fig. Chemicals used in this study.** (A) BADAN and its bioconjugation to E166C β-lactamase via the formation of a thiol ester bond to give E166Cb; (B) β-lactam antibiotics.
