# Supplementary material for: BADAN-conjugated β-lactamases as biosensors for β-lactam antibiotic detection
Source: PLoS One. 2020 Oct 30;15(10):e0241594. doi: 10.1371/journal.pone.0241594 (PMC7598492; doi:10.1371/journal.pone.0241594)
Supplement: S2 Fig — Peak A refers to the major species of the sample. E166Cb: calculated mass = 72778 Da and measured mass = 72778.48 ± 1.01 Da; E166Cb/N170Q: calculated mass = 72792 Da and measured mass = 72792.4 ± 3.78 Da. Calculated masses of E166Cb and E166Cb/N170Q were deduced from the sequence of the corresponding protein with the addition of one BADAN molecule. (DOCX) [file pone.0241594.s002.docx]

**
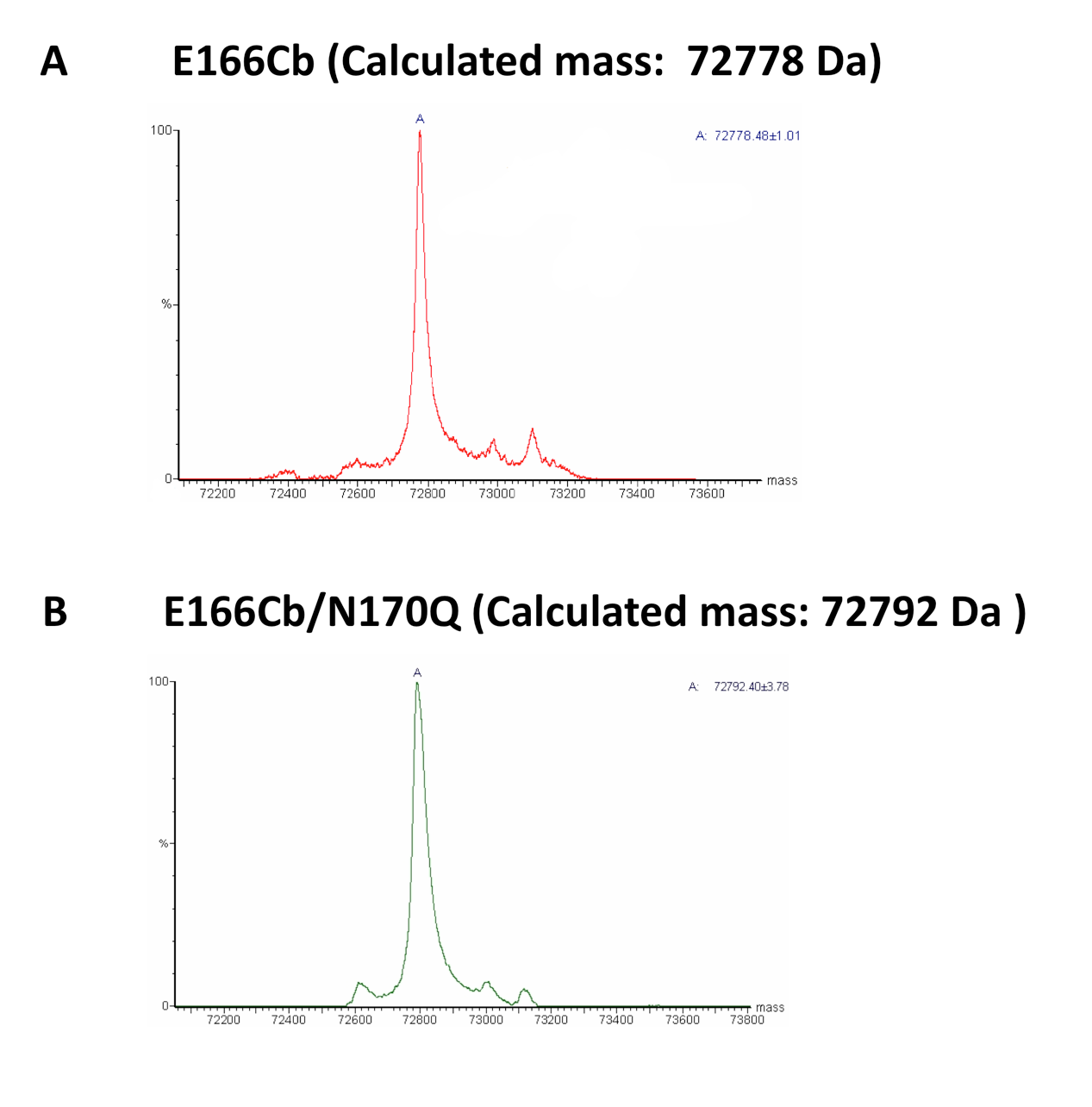
**

**S2 Fig. Mass spectra of (A) E166Cb and (B) E166Cb/N170Q.** Peak A refers to the major species of the sample. E166Cb: calculated mass = 72778 Da and measured mass = 72778.48 ± 1.01 Da; E166Cb/N170Q: calculated mass = 72792 Da and measured mass = 72792.4 ± 3.78 Da. Calculated masses of E166Cb and E166Cb/N170Q were deduced from the sequence of the corresponding protein with the addition of one BADAN molecule.
