# Supplementary material for: BADAN-conjugated β-lactamases as biosensors for β-lactam antibiotic detection
Source: PLoS One. 2020 Oct 30;15(10):e0241594. doi: 10.1371/journal.pone.0241594 (PMC7598492; doi:10.1371/journal.pone.0241594)
Supplement: S3 Fig — Red line: before labeling; Green line: after labeling. (DOCX) [file pone.0241594.s003.docx]

**
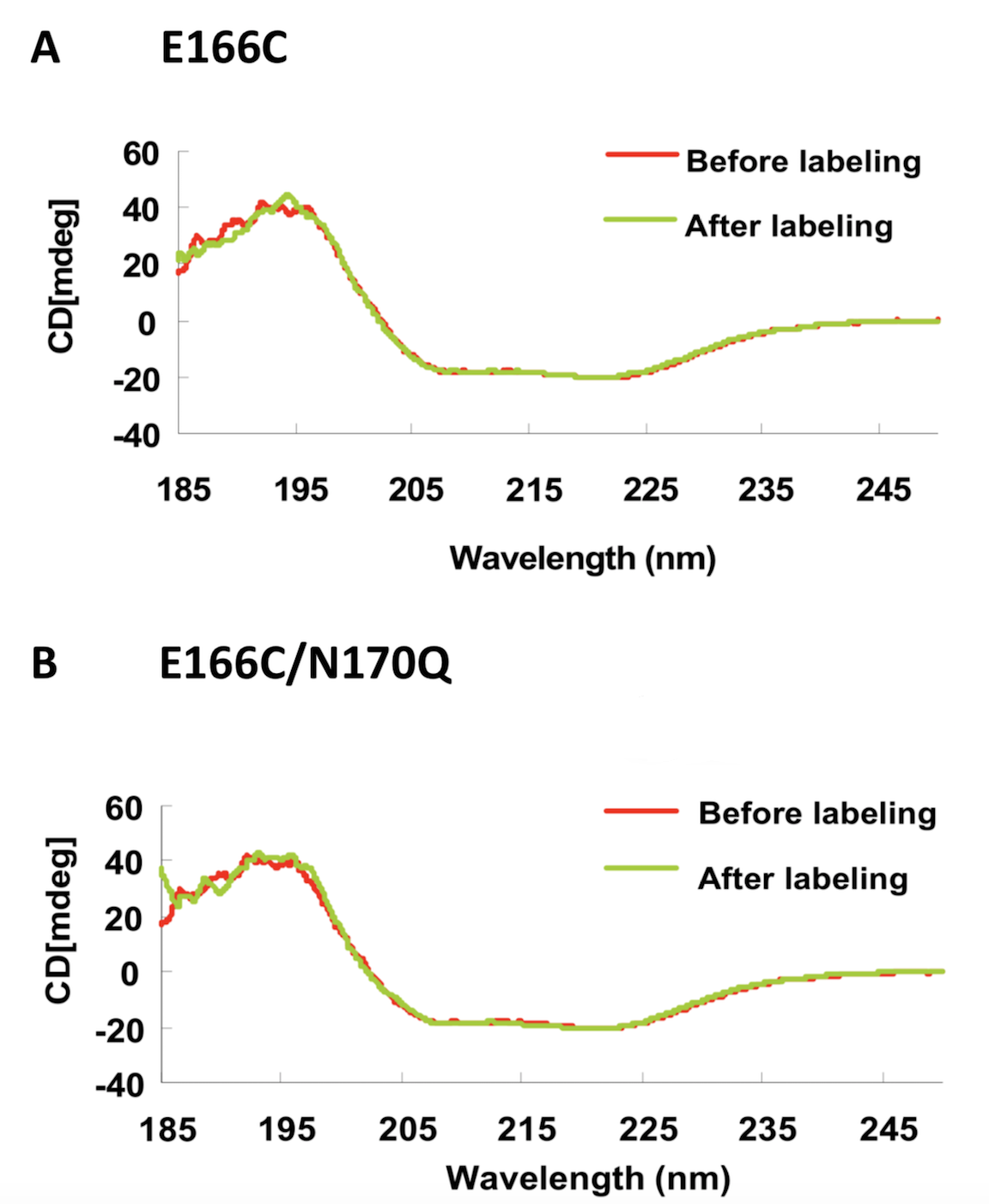
**

**S3 Fig. Far-UV CD spectra of (A) E166C and (B) E166C/N170Q before and after labeling with BADAN.** Red line: before labeling; Green line: after labeling.
