# Supplementary material for: BADAN-conjugated β-lactamases as biosensors for β-lactam antibiotic detection
Source: PLoS One. 2020 Oct 30;15(10):e0241594. doi: 10.1371/journal.pone.0241594 (PMC7598492; doi:10.1371/journal.pone.0241594)
Supplement: S4 Fig — (A) Root-mean-square deviation (RMSD) calculated using the energy-minimized structure after NVT and NPT equilibrations (npt8.gro) as the reference; (B) Radius of gyration (Rg); Red line: apo-E166Cb; Blue line: penicillin G-bound E166Cb. (DOCX) [file pone.0241594.s004.docx]

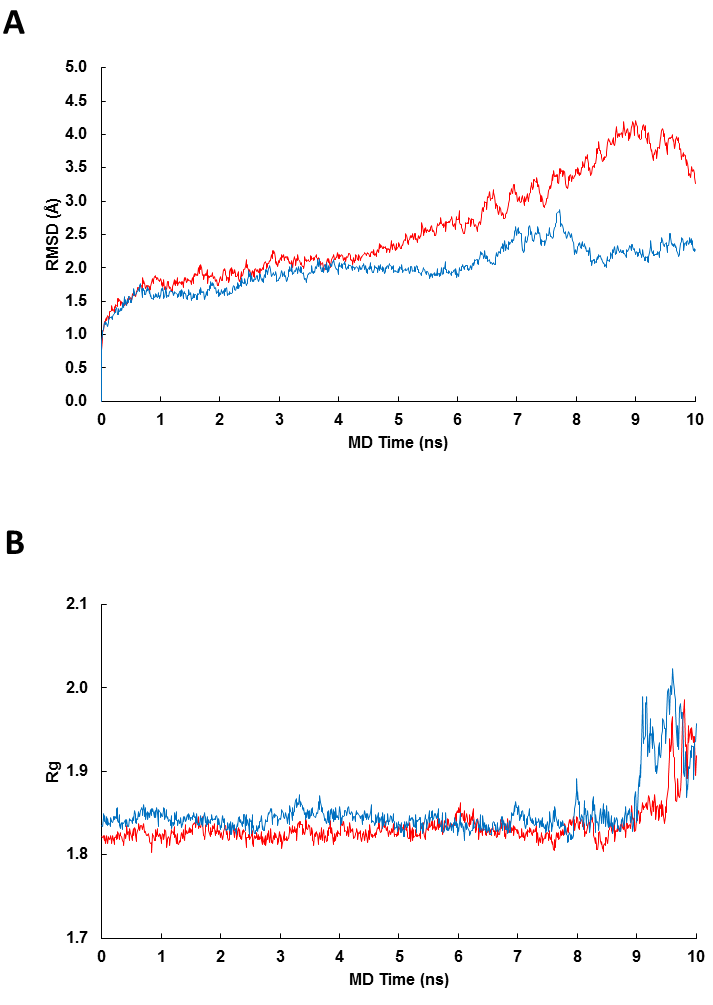


**S4 Fig. Properties of the protein structure in the MD stimulation.** (A) Root-mean-square deviation (RMSD) calculated using the energy-minimized structure after NVT and NPT equilibrations (npt8.gro) as the reference; (B) Radius of gyration (Rg); Red line: apo-E166Cb; Blue line: penicillin G-bound E166Cb.
