# Supplementary material for: BADAN-conjugated β-lactamases as biosensors for β-lactam antibiotic detection
Source: PLoS One. 2020 Oct 30;15(10):e0241594. doi: 10.1371/journal.pone.0241594 (PMC7598492; doi:10.1371/journal.pone.0241594)
Supplement: S5 Fig — (A) Accessible surface area of BADAN; (B) Distance of the mass center between BADAN and Tyr-105; Red line: apo-E166Cb; Blue line: penicillin G-bound E166Cb. Error bar of each data point represents the mean ± standard derivation. (DOCX) [file pone.0241594.s005.docx]

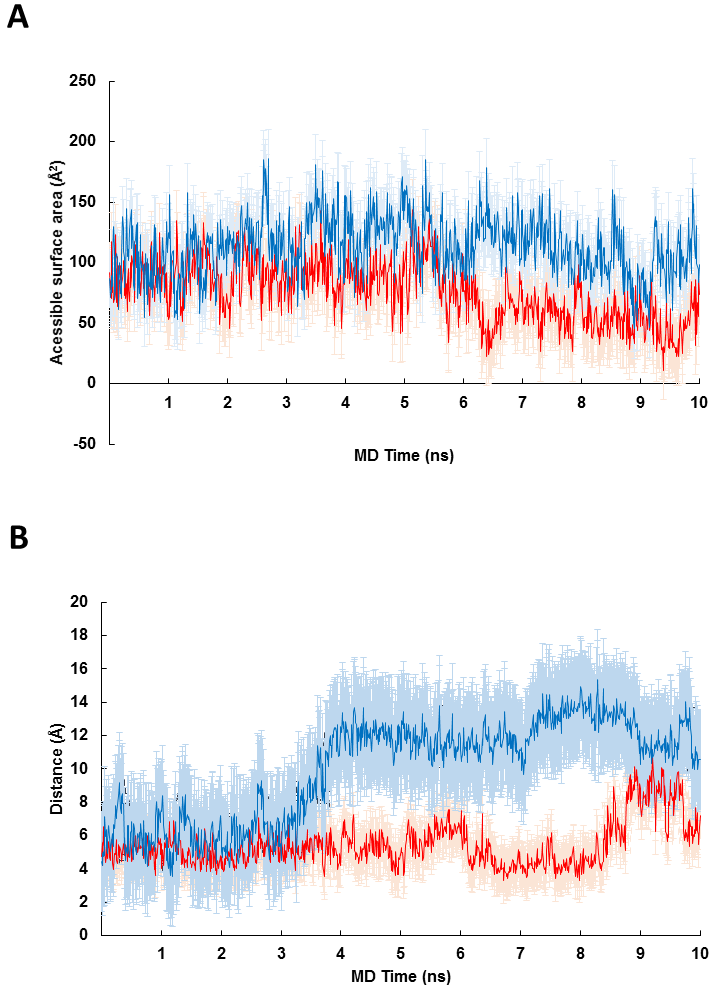


**S5 Fig. Properties of BADAN in the MD trajectories.** (A) Accessible surface area of BADAN; (B) Distance of the mass center between BADAN and Tyr-105; Red line: apo-E166Cb; Blue line: penicillin G-bound E166Cb. Error bar of each data point represents the mean ± standard derivation.
