# Supplementary material for: BADAN-conjugated β-lactamases as biosensors for β-lactam antibiotic detection
Source: PLoS One. 2020 Oct 30;15(10):e0241594. doi: 10.1371/journal.pone.0241594 (PMC7598492; doi:10.1371/journal.pone.0241594)
Supplement: S6 Fig — (A) Structures of penicillin and cephalosporin; (B) An overlay of penicillin G and cephaloridine in the active site of E166Cb. Active site Ser-70: cyan; BADAN: green; penicillin G: red; Cephaloridine: yellow. (DOCX) [file pone.0241594.s006.docx]

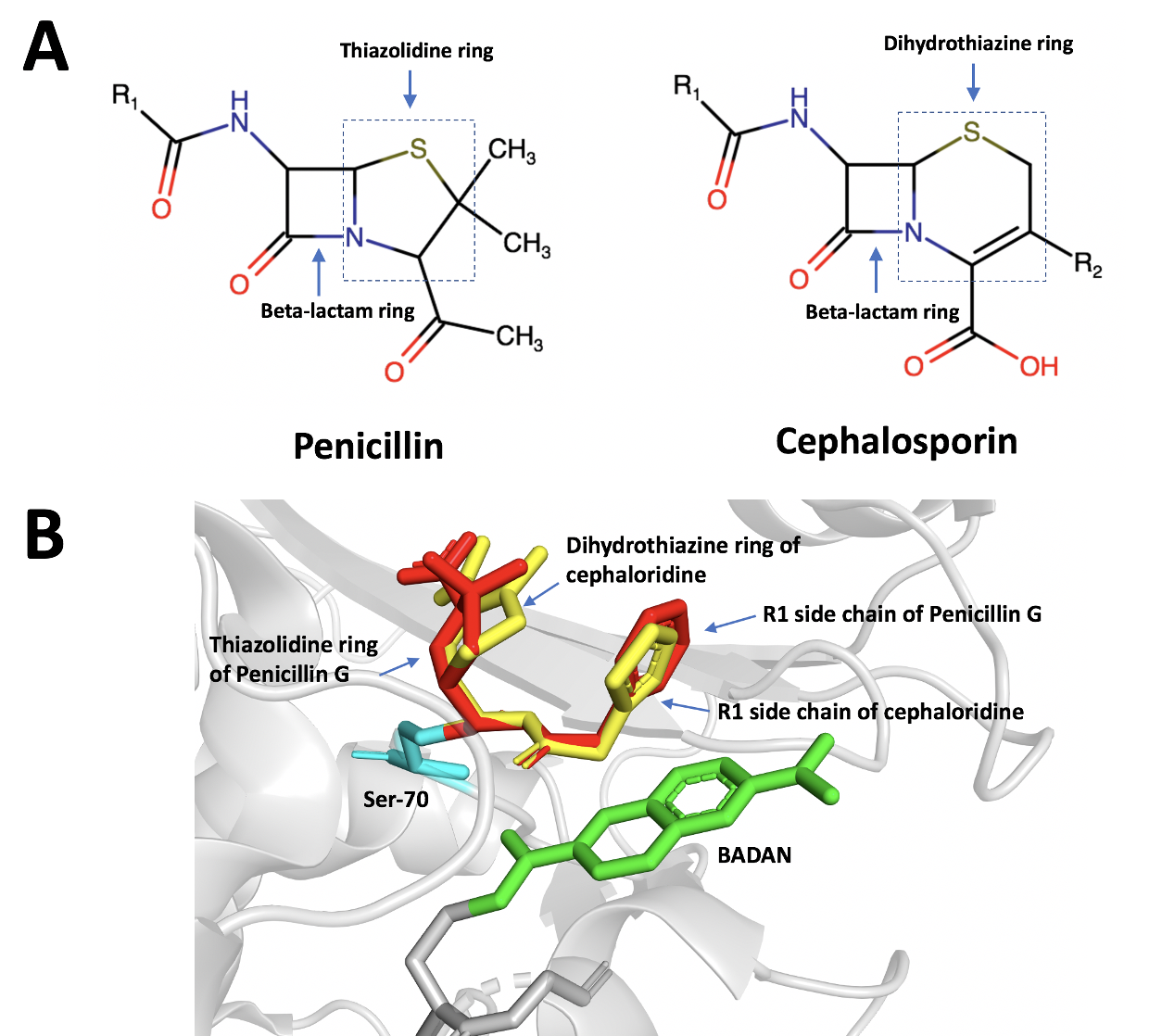


**S6 Fig. The substrate-bound models of E166Cb with either penicillin G and cephaloridine.** (A) Structures of penicillin and cephalosporin; (B) An overlay of penicillin G and cephaloridine in the active site of E166Cb. Active site Ser-70: cyan; BADAN: green; Penicillin G: red; Cephaloridine: yellow.
