# Supplementary material for: BADAN-conjugated β-lactamases as biosensors for β-lactam antibiotic detection
Source: PLoS One. 2020 Oct 30;15(10):e0241594. doi: 10.1371/journal.pone.0241594 (PMC7598492; doi:10.1371/journal.pone.0241594)
Supplement: S7 Fig — (A) Penicillin G; (B) Penicillin V; (C) Cefotaxime; (D) Moxalactam; E166Cb: red line; E166Cb/N170Q: blue line; E166Cf: green line; E166Cf/N170Q: cyan line. Curves of E166Cf and E166Cf/N170Q were adapted from our previous data [22] (https://pub.acs.org/doi/10.1021/acsomega.9b02211) and reprinted in part with permission from American Chemical Society (ACS). A further permission of this data should be directed to the ACS. (DOCX) [file pone.0241594.s007.docx]

**
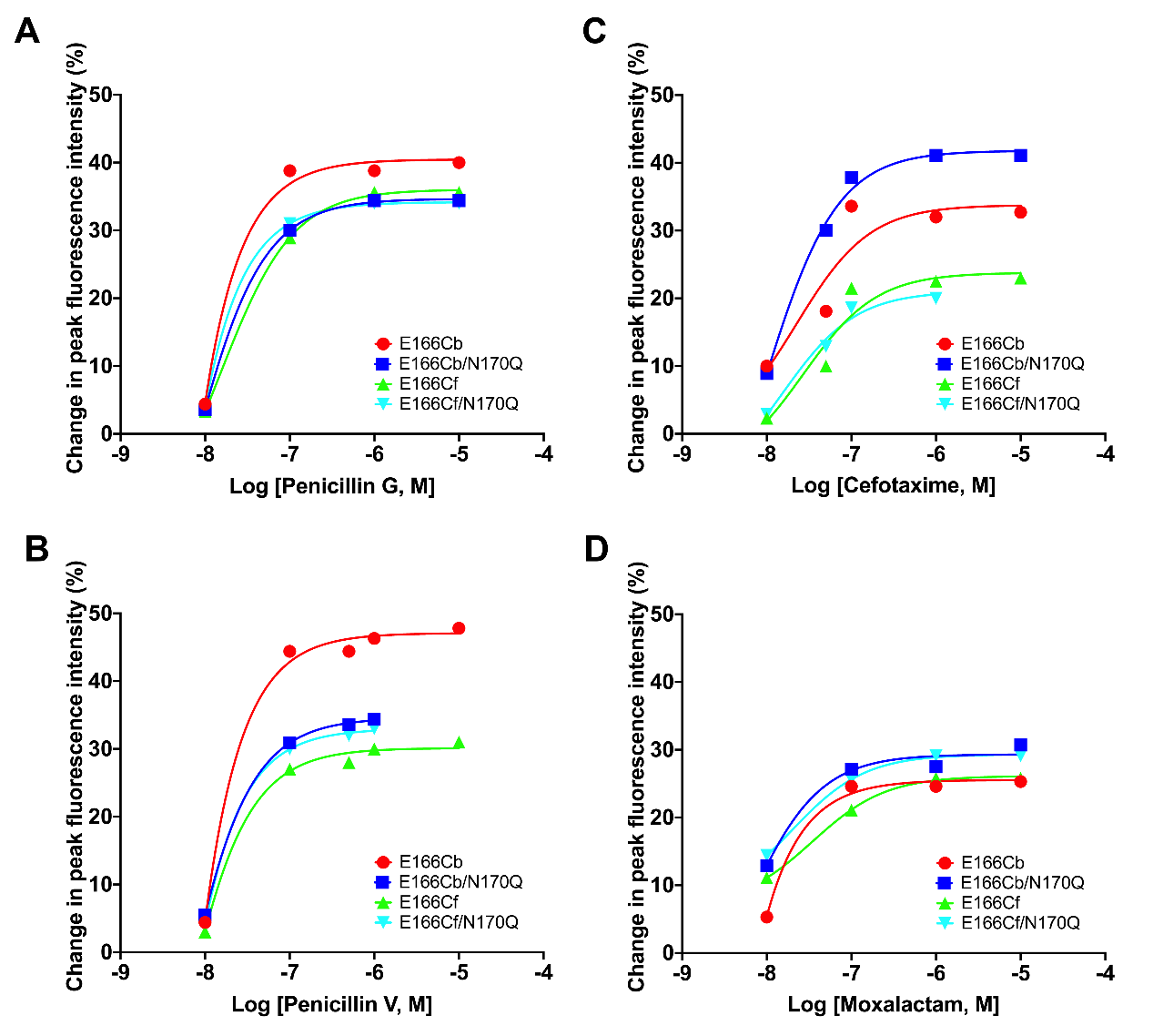
**

**S7 Fig. Calibration curves of β-lactam antibiotic detection.** (A) Penicillin G; (B) Penicillin V; (C) Cefotaxime; (D) Moxalactam; E166Cb: red line; E166Cb/N170Q: blue line; E166Cf: green line; E166Cf/N170Q: cyan line. Curves of E166Cf and E166Cf/N170Q were adapted from our previous data [22] (https://pub.acs.org/doi/10.1021/acsomega.9b02211) and reprinted in part with permission from American Chemical Society (ACS). A further permission of this data should be directed to the ACS.
